# Three-terminal heterojunction bipolar transistor solar cell for high-efficiency photovoltaic conversion

**DOI:** 10.1038/ncomms7902

**Published:** 2015-04-22

**Authors:** A. Martí, A. Luque

**Affiliations:** 1 Instituto de Energía Solar, Universidad Politécnica de Madrid, ETSI Telecomunicación, Ciudad Universitaria sn, Madrid 28040, Spain

## Abstract

Here we propose, for the first time, a solar cell characterized by a semiconductor transistor structure (n/p/n or p/n/p) where the base–emitter junction is made of a high-bandgap semiconductor and the collector is made of a low-bandgap semiconductor. We calculate its detailed-balance efficiency limit and prove that it is the same one than that of a double-junction solar cell. The practical importance of this result relies on the simplicity of the structure that reduces the number of layers that are required to match the limiting efficiency of dual-junction solar cells without using tunnel junctions. The device naturally emerges as a three-terminal solar cell and can also be used as building block of multijunction solar cells with an increased number of junctions.

## Abstract (web summary)

Multiple junction solar cells offer the means to high-efficiency photovoltaics but suffer from complicated manufacturing and packing. Here Martí *et al*., propose a three-terminal heterojuntion bipolar transistor solar cell that simplifies the structure reducing the number of layers while maintaining the efficiency.

## Graphical abstract

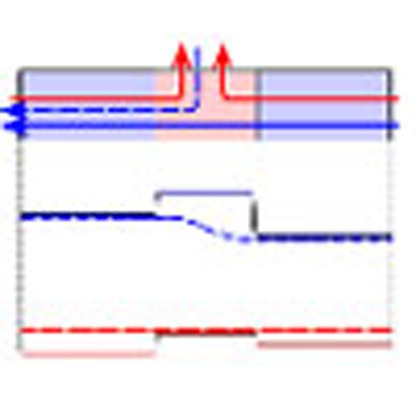

The use of several semiconductors of different bandgaps to make a better use of the solar spectrum for photovoltaic energy conversion was first proposed by Jackson^1^ in 1958. Wolf^2^, in 1960, pointed out the difficulty in using tandem solar cells based on (p/n)–(p/n) semiconductor junctions to take to practice this approach because of the impossibility of having electrical current circulating across a p/n junction biased in reverse. The problem could be solved thanks to tunnel diodes (a highly doped p^++^–n^++^ junction), invented by Esaki^3^ and nowadays incorporated to multijunction solar cells^4^,^5^,^6^. More recently, a ‘wafer bonding' scheme has been developed^7^ to attach different solar cells each one consisting of a (p/n) junction.

In this work we propose the use of p/n/p (or n/p/n) structures instead, that exhibit the same limiting efficiency that a dual-junction solar cell, but without the need of using tunnel junctions or wafer bonding schemes for interconnecting the cells. The proposed solar cell structure has three-terminals. Three- or more- terminal solar cell structures have been proposed in the past, but consisted either in a mechanical stack of single-gap solar cells^8^ or included a doped semiconductor layer to interconnect the solar cells in a (n/p)–p–(n/p)^9^ or (n/p)–p–(p/n)^10^ configuration.

The structure we propose looks similar to the one of a bipolar junction transistor^11^ (BJT). The performance of a BJT is described in terms of its ‘transport factor' (*α*_T_) and ‘emitter injection efficiency' (*γ*). In order a BJT to approach its ideal performance, *α*_T_→1 and *γ*→1. However, as we shall see, for the ideal performance of the solar cell structure we propose *γ*→0.

## Results

### Description of the structure

**Figure 1:**
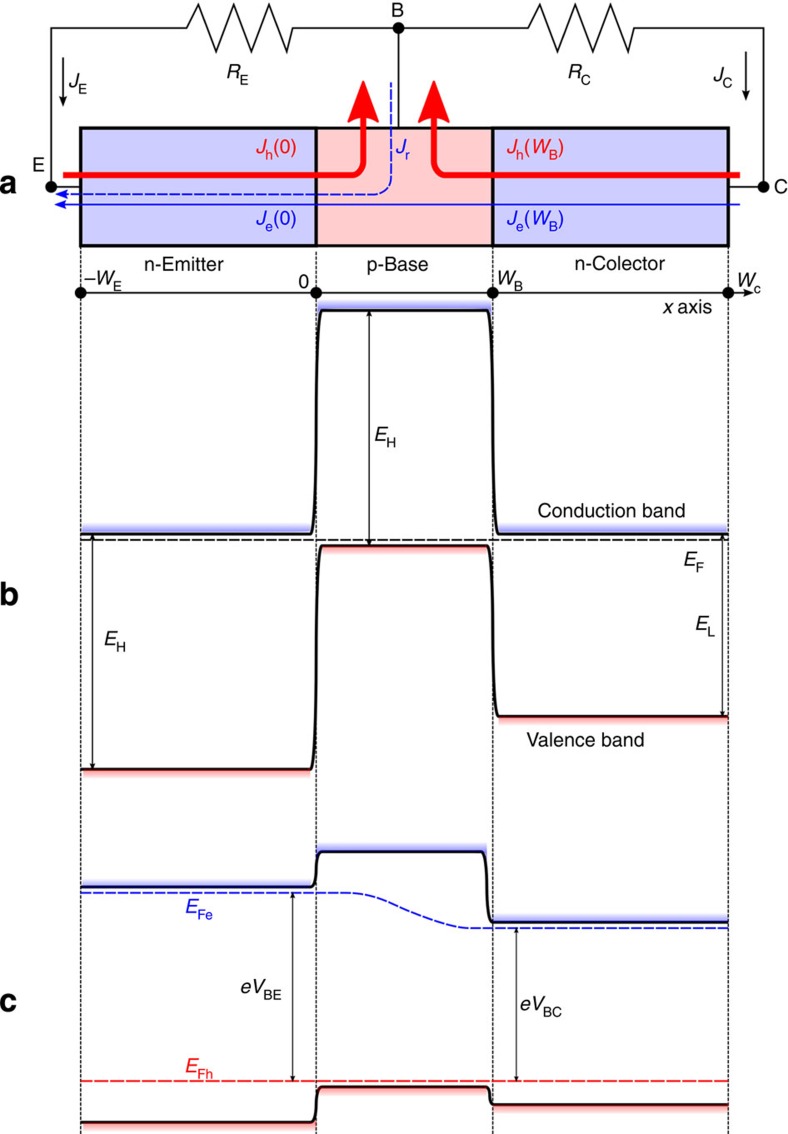
Structure of the three-terminal heterojunction bipolar transistor solar cell. Simplified layer structure showing also electron (blue) and hole (red) current densities with sign criterion that current densities are positive when flowing in the direction indicated by the arrow (**a**). Simplified bandgap diagram in equilibrium (**b**). Simplified diagram under conditions in which the cell is delivering electrical power (**c**). *E*_Fe_ and *E*_Fh_ are the electron and hole quasi-Fermi levels.

Figure 1 shows the basic structure and simplified bandgap diagram of the three-terminal heterojunction bipolar transistor solar cell (HBTSC) that we propose. We will assume an *npn* structure but the same analysis trivially applies to a *pnp* structure by swapping the role of electrons and holes. Arrows indicating the direction that electron and hole current densities have through the base–emitter and base–collector junctions (when assumed positive) are also indicated. Anticipating final results, the directions of the currents have been chosen in a way that they will all become positive under normal cell operation. The base–emitter junction, which is the first facing the sun, is made of a high-bandgap semiconductor (designated as *E*_H_), and the collector is made of a low-bandgap semiconductor (designated as *E*_L_). The base–emitter and the base–collector junction area will be assumed equal and equal to area of the cell, *A*. Once the theory presented here is understood, it can be easily modified for accounting for differences in these areas.

### Model and limiting efficiency

To apply Shockley–Queisser detailed-balance^12^ theory to the study of the limiting efficiency of this solar cell, we will assume that carrier generation-recombination processes only occur through the absorption and emission of photons (radiative recombination). For simplification, the sun will be assumed as a black body at the temperature *T*_S_=6,000 K and the cell at the ambient temperature *T*_C_=300 K. Assuming the sun as a black body at 6,000 K has become de facto a standard for calculating the limiting efficiency of new proposed devices, because it allows comparing easily the efficiency limit of one proposal against another.

As it is characteristic of an ideal transistor structure^13^, the carrier transport factor through the base will be assumed one (*α*_T_=1). For the moment, readers can exemplify this feature by assuming the base of the transistor as being very short so that the total generation and recombination of carriers in this region becomes negligible. In the plot in Fig. 1 this will imply that the electron recombination current *J*_r_ will be assumed zero. We will later lift this restriction.

We calculate first the hole current density crossing the base–emitter junction, *J*_h_(0). For that, by applying the continuity equation to holes in the emitter, we find:


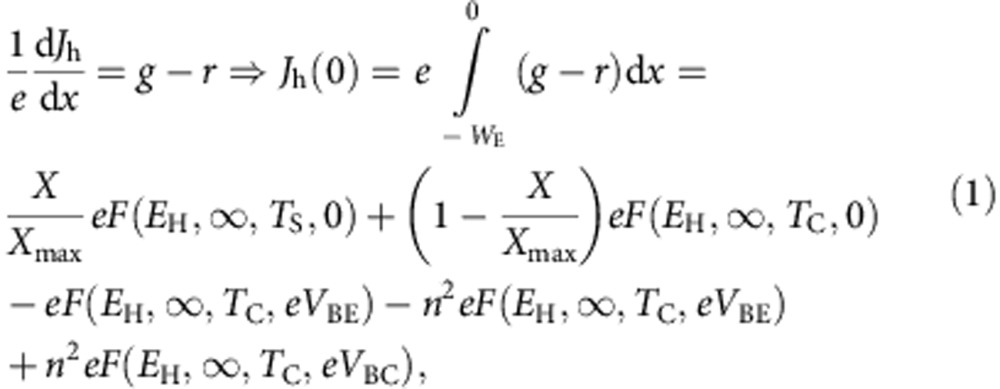


where: *g* and *r* are the electron-hole generation and recombination rates, respectively; *e* is the electron charge; *X* is the concentration of solar light; *X*_max_=46,050 is the maximum concentration when the cell is surrounded by air; and


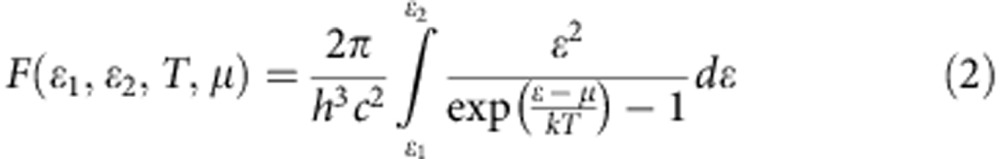


being *h* the Planck's constant, *c* the speed of light in vacuum and *k* the Boltzman's constant^14^. From a physical point of view, *F* corresponds to a photon flux, characterized by the temperature *T* and chemical potential *μ*, and with energy in between *ɛ*_1_ and *ɛ*_2_.

**Figure 2:**
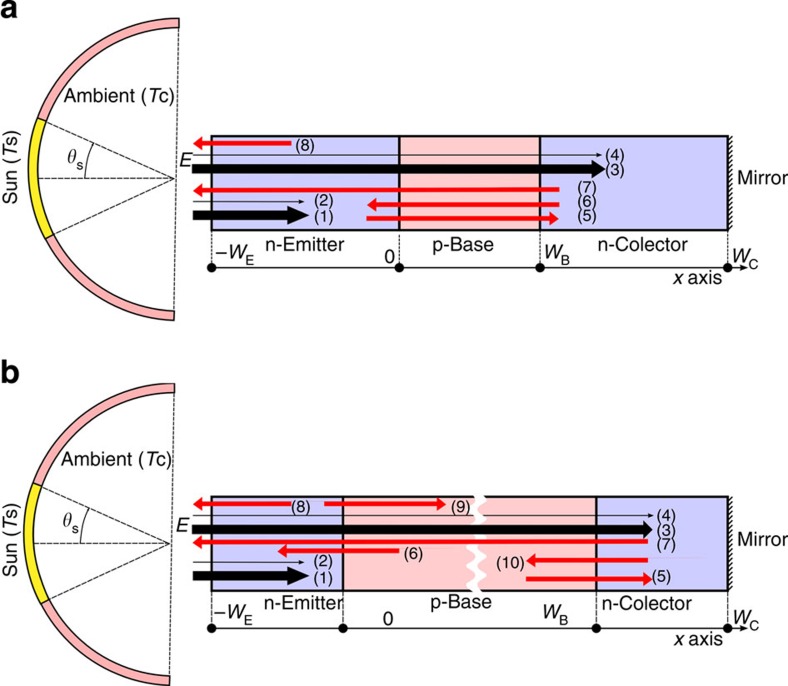
Illustration of the photon fluxes involved in the operation of HBTSC. (**a**) illustrates the case when the base is short and (**b**) the case when the base is long. Black arrows represent photon fluxes from the sun and red arrows represent electroluminescent photons.

In the calculations leading to the results in equation (1) we have assumed that: (a) the emitter has an absorptivity that equals one for photons with energy higher than *E*_H_; (b) the front face of the emitter, located at *W*_E_, is passivated so that *J*_h_(−*W*_E_)=0; (c) carrier mobility is large so that the split of electron and hole quasi-Fermi levels is constant through the emitter and equal to the voltage applied to the base–emitter junction, *V*_BE_ (in eV units) and; (d) the front of the cell is surrounded by air (refraction index one) and all the semiconductors involved in the structure have a refraction index *n*; e) the cell is illuminated through the emitter by the sun and the ambient and a back reflector prevents luminescent radiation from escaping through the rear side. With these hypotheses, equation (1) has been obtained following the same method described in ref. 15. In the last line of equation (1), 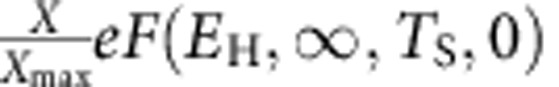 corresponds to the photons absorbed from the sun (represented by arrow number 1 in Fig. 2). *X*=sin^2^*θ*/sin^2^*θ*_S_ (being *θ* the semiangle of the solar disc seen from Earth after a concentrator system is used and *θ*_S_≈0.267° being the same angle when no concentrator is used) represents the solar concentration. *X*_max_=46,050 represents the maximum theoretical concentration when the media surrounding the cell is air and is obtained for *θ*=*π*/2. *T*_*S*_ is the temperature of the sun (assumed as 6,000 K).

The term 
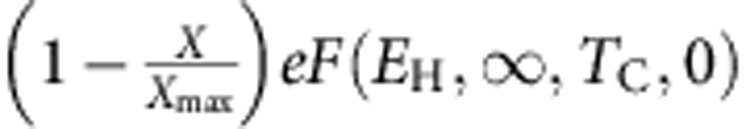
 corresponds to the photons absorbed from the thermal surroundings (represented by arrow number 2 in Fig. 2) being *T*_C_=300 K the ambient temperature.

The term *eF(E*_H_, ∞, *T*_C_, *eV*_BE_) corresponds to the electroluminescent photons emitted by the emitter through its front surface (represented by arrow number 8 in Fig. 2).

The term *n*^2^*eF(E*_H_, ∞, *T*_C_, *eV*_BE_) corresponds to the photons emitted by the emitter through its back surface. Since the base is short, photons emitted through the emitter back surface will entirely reach the collector (represented by arrow number 5 in Fig. 2).

The term *n*^2^*eF(E*_H_, ∞, *T*_C_, *eV*_BC_) corresponds to photons absorbed at the emitter due to the electroluminescent emission from the collector (represented by arrow number 6 in Fig. 2). Due to a short base, photons emitted by the collector with energy higher than *E*_H_ are absorbed at the emitter.

The collector will be assumed to have also absorptivity equal to one, so none of the photons that reach the collector due to the emitter electroluminescence return to the emitter. Notice that, in equilibrium, *T*_S_=*T*_C_ and *J*_h_(0)=0 as expected.

The total current density through the emitter–base junction and, therefore, the total current density through the emitter terminal will be then:


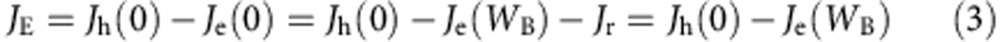


where the hole current density, *J*_h_(0), is given by equation (1) (*J*_r_ being assumed zero) and the electron current density *J*_e_(*W*_B_) remains to be calculated.

We calculate now the hole current density through the base–collector junction. Applying the continuity equation for holes at the collector, we find that, with the sign criteria adopted in Fig. 1:


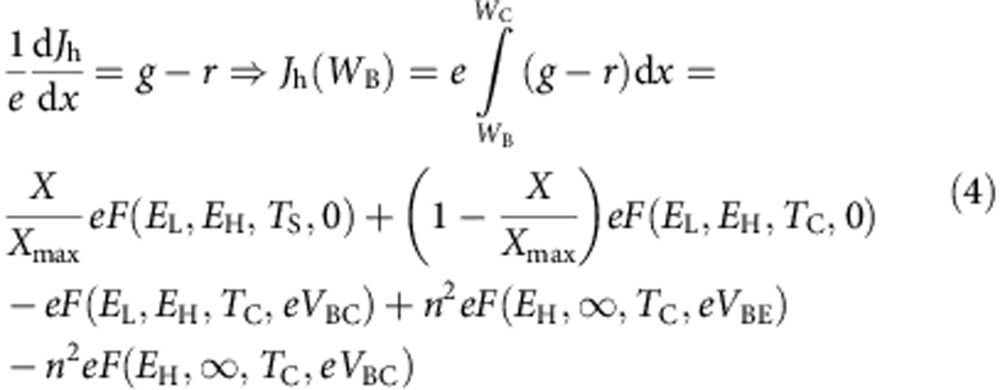


For this calculation we have assumed similar conditions to the ones we assumed for the emitter: (a) the collector has an absorptivity that equals one for photons with energy higher than *E*_*L*_; (b) the rear face of the collector, located at *x=W*_C_, is passivated so that *J*_h_(*W*_C_)=0; (c) carrier mobility is large so that the split of electron and hole quasi-Fermi levels is constant through the collector and equal to the voltage applied to the base–collector junction, *V*_BC_; (d) the front of the cell is surrounded by air (refraction index one) and all the semiconductors involved have a refraction index *n*; and (e) the cell has a back reflector so that there is no luminescent emission through the rear side of the cell.

In the last line of equation (4), 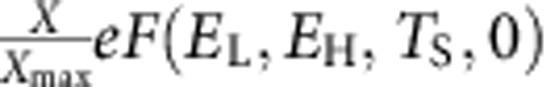 corresponds to the photons absorbed from the sun at the collector (represented by arrow number 3 in Fig. 2). The term 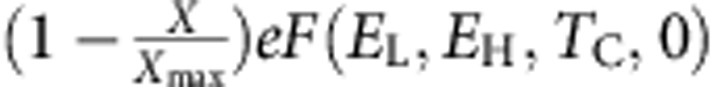 corresponds to the photons absorbed from the thermal surroundings at the collector (represented by arrow number 4 in Fig. 2).

The term *eF(E*_L_, *E*_H_, *T*_C_, *eV*_BC_) corresponds to the electroluminescent photons emitted by the collector through its front surface (represented by arrow number 7 in Fig. 2). Notice the emitter is transparent to these photons because of its higher bandgap.

The term *n*^2^*eF(E*_H_, ∞, *T*_C_, *eV*_BE_) corresponds to the photons emitted by the emitter through its back surface and absorbed at the collector. Since the base is short, photons emitted through the emitter back surface entirely reach the collector (represented by arrow number 5 in Fig. 2).

The term *n*^2^*eF(E*_H_, ∞, *T*_C_, *eV*_BC_) corresponds to the photons absorbed at the emitter due to the electroluminescent emission from the collector (represented by arrow number 6 in Fig. 2).

The total current density through the base–collector junction and, therefore, the total current density through the collector terminal will be then:


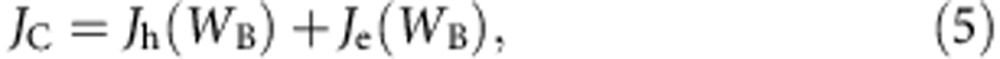


where the hole current density, *J*_h_(*W*_B_), is given by equation (4) and, as mentioned, the electron current density *J*_e_(*W*_B_) remains to be calculated.

We calculate next the electrical power, *P*, extracted from the cell per unit of area. This power is extracted through two resistors *R*_E_ and *R*_C_ attached to the base–emitter and base–collector terminals:


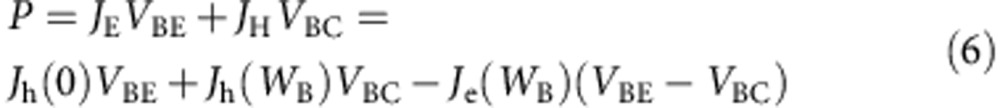


The term *J*_e_(*W*_B_)(*V*_BE_–*V*_BC_) deserves now our attention. As it will be proven next, it is always positive so that it represents a power-loss mechanism. Its physical origin is the entropy rate generated by the electron current density flowing in the direction of decreasing slope of the electron-quasi-Fermi level in the base. If, as plotted in Fig. 1, *V*_BE_>*V*_BC_, electrons (considered as particles) will flow from left to right and the electron current density *J*_e_(*W*_B_), with the sign criteria adopted in Fig. 1, will be positive (notice electron current density has opposite sign to the direction that electrons, as particles, actually flow). If we assume working conditions in which *V*_BE_<*V*_BC_, we will also have *J*_e_(*W*_B_)(*V*_BE_–*V*_BC_)>0 because then *J*_e_(*W*_B_)<0. Therefore, even if we do not calculate *J*_e_(*W*_B_), it has to be made equal to zero to maximize the efficiency of the cell. In practice, this will demand doping the base at a much higher level than the doping of the emitter for the cases in which *V*_BE_>*V*_BC_ and much higher than the collector for the cases in which *V*_BE_<*V*_BC_. Since we anticipate that, when the cell delivers its maximum power, we will have *V*_BE_>*V*_BC_, making the base doping higher than the emitter doping should play the major role of the design. In bipolar transistor terminology, this implies that the emitter injection efficiency (the ratio between the electron and the total emitter current densities crossing the emitter–base junction) has to be as close to zero as possible. In this respect, the design of the HBTSC differs from the design of a conventional bipolar transistor, where emitter injection efficiencies as close to one as possible are seek.

If *J*_e_(*W*_B_)=0, it can be easily checked that the model describing *J*_E_ and *J*_C_ is exactly the same one than that of dual-junction solar cell independently connected^16^, where *J*_E_ would describe the current–voltage characteristic of the top cell and *J*_C_ would describe the current–voltage characteristic of the bottom cell. The only conceptual difference is that, in the work in ref. 16, the media coupling the cells was air or none—if a selective mirror was inserted in between the cells—while here the cells are optically coupled through the semiconductor refraction index. Therefore, the HBTSC and the dual-junction solar cell share the same limiting efficiency.

### Impact of the bandgaps and injection efficiency

**Figure 3:**
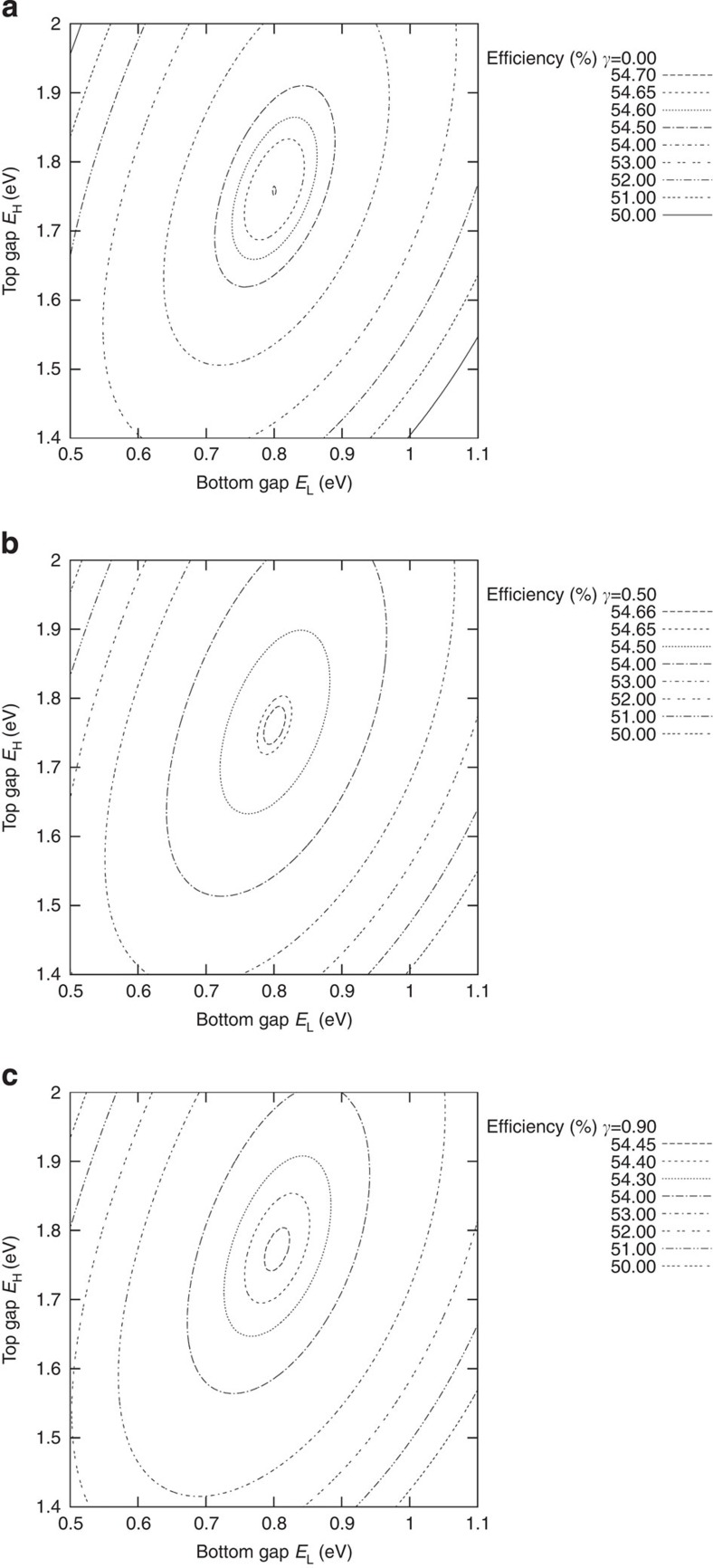
Efficiency limit of the three-terminal heterojunction bipolar solar cell. Contour plots showing the limiting efficiency of the HBTSC as a function of the top and bottom bandgaps when operated at maximum concentration and for several injection efficiencies: (**a**) *γ*=0, (**b**) *γ*=0.5 and (**c**) *γ*=0.9.

In Fig. 3 we plot the limiting efficiency of the cell as a function of the top and bottom bandgaps. Maximum efficiency (54.7%) is obtained for *E*_L_*=*0.8 eV and *E*_H_*=*1.76 eV.

We include plots also for the cases in which *J*_e_(*W*_B_)≠0. For a short base, the minority carrier excess in the base can be approached by a linear function and, assuming low-injection conditions in the base:


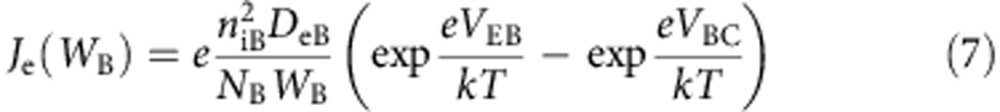


where *n*_iB_^2^ is the intrinsic concentration of the base semiconductor, *N*_B_ is the base doping and *D*_eB_ is the diffusion constant of the electrons at the base. The plots are then represented for several values of the base–emitter injection efficiency, defined (as in a bipolar transistor) as:


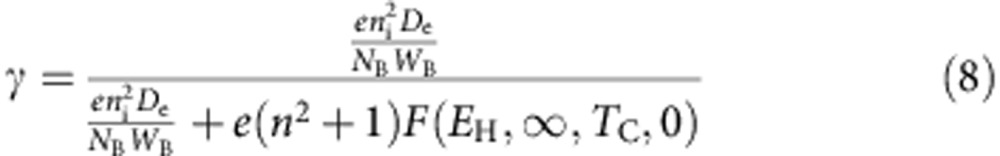


where *e(n*^2^+1)*F(E*_H_, ∞, *T*_C_, 0) represents the reverse saturation current of the holes crossing the base–emitter junction.

As anticipated, the impact of *γ*≠0 is to reduce the limiting efficiency of the cell. However, limiting efficiencies above 50% are preserved for base–emitter injection efficiencies as high as 0.9. Keeping injection efficiencies much lower than this value should not imply any technological challenge by increasing the base doping (what justifies also our initial hypothesis of a base operating in low-injection regimen). On the other hand, there is no apparent shift of the optimum gaps as *γ* is increased.

### Current–voltage characteristics

**Figure 4:**
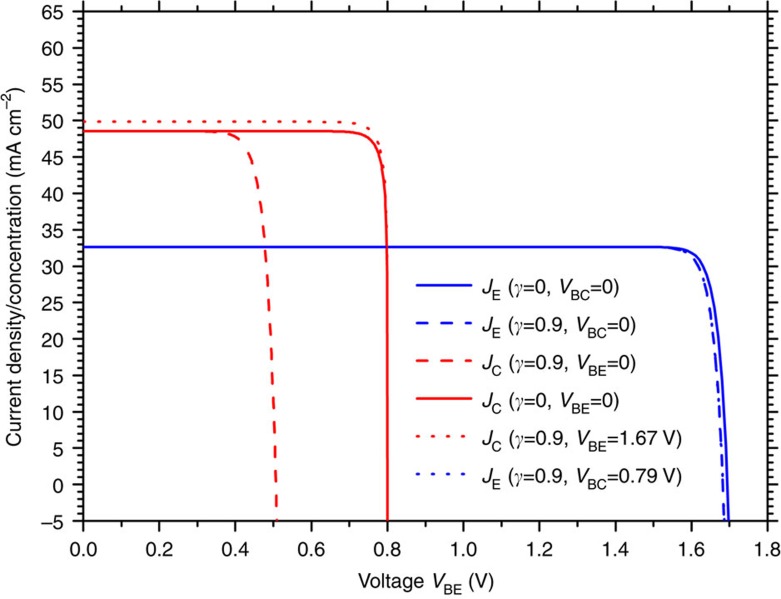
Current density–voltage characteristics. Emitter and collector current density–voltage characteristics of the HBTSC for several values of the injection efficiency (□) and different polarization of the base–emitter and base–collector junctions. The modelled cell is characterized by the following bandgaps: *E*_H_=1.76 eV and *E*_L_=0.8 eV. The results correspond to the maximum concentration case (46,050 suns).

To gain further insight into the solar cell operation, Fig. 4 plots the current density–voltage characteristic of the solar cell (both for the emitter and the collector currents), for optimum bandgaps and maximum solar concentration case and several working conditions. The comparison between the plots corresponding to the cases *γ*=0 and *γ*=0.9, for example, allows visualizing in the curves the impact of *γ* as loss factor. On the other hand, comparison of the collector current between the cases *J*_C_(*γ*=0.9, *V*_BE_=0) and *J*_C_(*γ*=0.9, *V*_BE_=1.67 V) reveal an increment in the collector short-circuit photocurrent as *V*_BE_ increases due to the luminescent coupling between collector and emitter (the emitter shines more light into the collector when *V*_BE_=1.67 V than when *V*_BE_=0 V). This increment in the short-circuit current is also present in the emitter current (cases *J*_E_(*γ*=0.9, *V*_BC_=0) and *J*_C_(*γ*=0.9, *V*_BC_=0.7967 V)) but is barely perceived in the plot.

### Long base case

Although results above have been obtained under the approximation of a ‘short' base, the results are equally valid in the radiative limit for a ‘long' base. This is due to the photon-recycling phenomena that takes place in the base, that makes radiative total recombination independent on the base thickness since a photon that is emitted is likely recycled in a long base^17^ and therefore, its recombination will not count. In effect, when the base is ‘long', the photons emitted by the emitter by its rear side are fully absorbed in the base and do not reach the collector. Equally, the emitter receives through its base the photons emitted by the base and not by the collector (that are absorbed now instead in the base. The results in equation (1) must be modified obtaining instead (notice that, in the last term in equation (1), *V*_BC_ has been substituted now by *V*_BE_).


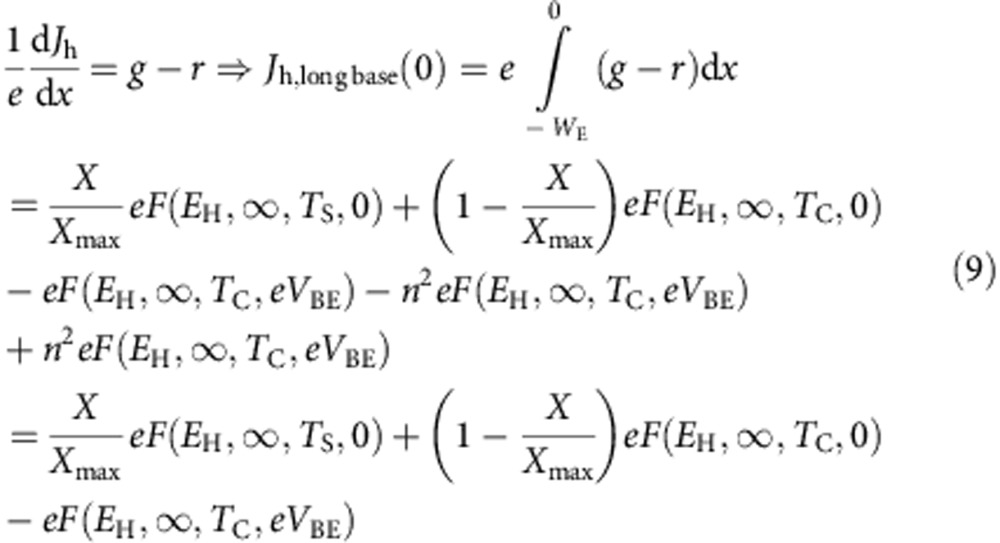


However, since the base is ‘long' we cannot assume now *J*_r_=0. If, as a worst case scenario (because recombination in the base reaches its maximum) at the time that a simplifying assumption, we assume that the electron and hole quasi-Fermi level split in the base equals *eV*_BE_ (the highest value) all along the base thickness, *J*_r_ can be calculated as:


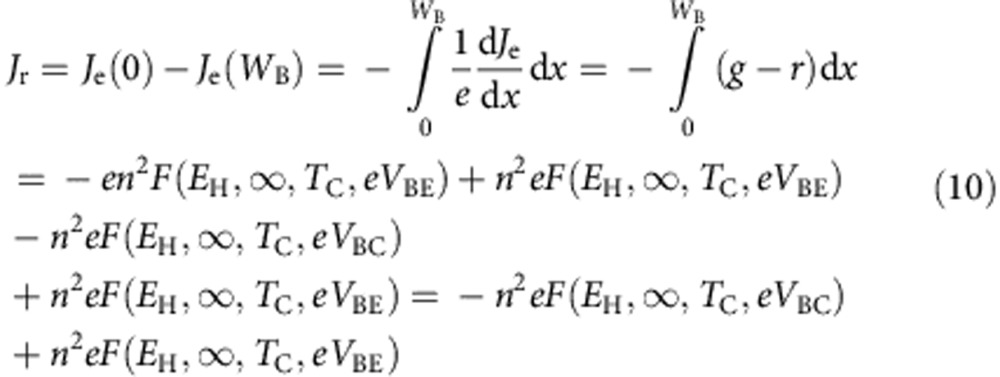


The emitter current now will be given by:


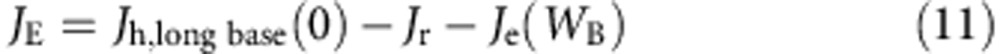


which taking into account equations (9) and (10) result equal to equation (3) obtained for the case the base was considered short. Therefore, the calculations made in the previous section remain the same.

## Discussion

**Figure 5:**
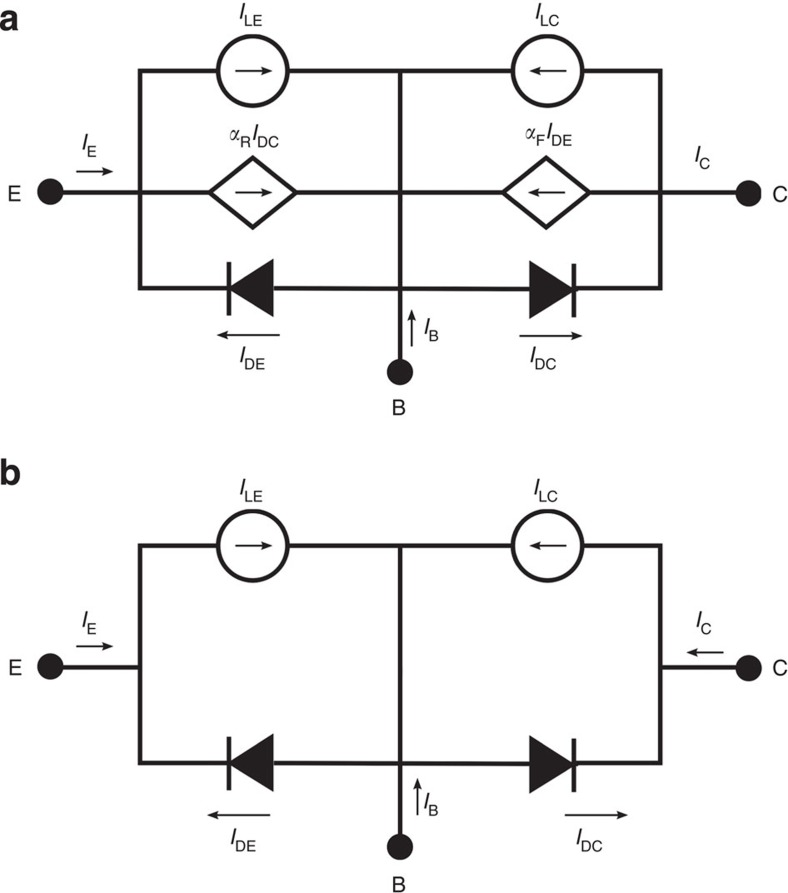
Circuital models. (**a**) Ebers–Moll model of the HBTSC. (**b**) The same model with the current gains in common base set to zero.

In a more general case, our model of the HBTSC can be visualized by the bipolar transistor Ebers–Moll model^13^ represented in Fig. 5a where *α*_F_ and *α*_R_ are the forward and reverse emitter and collector current gains in common base operation mode. The Ebers–Moll model has been modified to take into account for the current photogenerated by the junctions. In this respect, *I*_LE_ and *I*_LC_ correspond to the emitter and collector photogenerated currents, respectively, when the base–emitter and the base–collector junctions are short-circuited. If properly designed, photons with energy higher than the gap *E*_H_ will mostly contribute to *I*_LE_ while photons with energy higher than *E*_L_ will contribute only to *I*_LC_. If the transistor current gains *α*_F(R)_ are made zero, the model transforms into the model plotted in Fig. 5b. As it can be seen, in this model the emitter current, *I*_E_, and collector current *I*_C_, correspond now to the current–voltage characteristics of two independent solar cells. In agreement with the discussion involved in the detailed-balance limit above, *α*_F(R)_ will approach zero as the injection efficiency *γ* approaches zero.

In summary, we have proven that the detailed-balance limit of the HBTSC is the same than that of a dual-junction solar cell. Although the design of the cell reminds an heterojunction bipolar transistor^18^,^19^ there are conceptually important differences. First, in a heterojuntion bipolar transistor, the emitter injection efficiency *γ* has to be maximized (made equal to one); on the contrary, in our HBTSC, is has to be made equal to zero. Second, to approach *γ* to one, in the heterojunction bipolar transistor just the bandgap of the emitter is increased and not the bandgap of the base; in the HBTSC both the emitter and base are the regions with large bandgap.

Finally, we point out that the HBTSC can be considered as building block of more complex multiterminal multijunction solar. Hence, a four junction HBTSC could be built by piling up two HBTSCs, and so on. A tunnel junction between the two of these HBTSC would still not be required since there would not be current flow between one HBTSC and the next.

## Methods

Calculations in this work have been performed in Mathematica 5.0. Contour plots have been calculated with GnuPlot.

## Author contributions

A.M. and A.L. elaborated the concept. A.M. wrote the manuscript and performed the calculations.

## Additional information

**How to cite this article:** Martí, A. and Luque, A. Three-terminal heterojunction bipolar transistor solar cell for high-efficiency photovoltaic conversion. *Nat. Commun*. 6:6902 doi: 10.1038/ncomms7902 (2015).
